# Correction: Bilyy et al. Rapid Generation of Coronaviral Immunity Using Recombinant Peptide Modified Nanodiamonds. *Pathogens* 2021, *10*, 861

**DOI:** 10.3390/pathogens12121411

**Published:** 2023-11-30

**Authors:** Rostyslav Bilyy, Quentin Pagneux, Nathan François, Galyna Bila, Roman Grytsko, Yuri Lebedin, Alexandre Barras, Jean Dubuisson, Sandrine Belouzard, Karin Séron, Rabah Boukherroub, Sabine Szunerits

**Affiliations:** 1 Danylo Halytsky Lviv National Medical University, Pekarska Str., 69, 79010 Lviv, Ukraine; halyna.bila@gmail.com (G.B.); grytskoroman@gmail.com (R.G.); 2 University of Lille, CNRS, Centrale Lille, University Polytechnique Hauts-de-France, UMR 8520-IEMN, F-59000 Lille, France; quentin.pagneux@univ-lille.fr (Q.P.); alexandre.barras@univ-lille.fr (A.B.); rabah.boukherroub@univ-lille.fr (R.B.); 3 U1019-UMR 9017-CIIL-Center for Infection and Immunity of Lille, Institut Pasteur de Lille, University of Lille, CNRS, INSERM, CHU Lille, F-59000 Lille, France; nathan.francois@ibl.cnrs.fr (N.F.); jean.dubuisson@ibl.cnrs.fr (J.D.); sandrine.belouzard@ibl.cnrs.fr (S.B.); karin.seron@ibl.cnrs.fr (K.S.); 4 Xema Co., Ltd., Akademika Efremova Str., 23, 03179 Kyiv, Ukraine; lebedin@xema.com.ua

## Error in Figure

In the original publication [1], there was a mistake in Figure 2a as published. The TEM image of a modified ND rather than an ND itself is shown. The TEM image of the ND has been reported before and a link to the reference is provided. The corrected Figure 2a is shown below.

## Missing Citation

In the original publication, a Reference was not cited. This citation has now been inserted in Section 2.1, in the Figure 2 caption. Figure 2 caption should be “Characterization of nanodiamond particles (NDs) used in this work: (a) TEM (adapted with permission from ref. [32], 2015, Royal Society of Chemistry) and HR-TEM (inset), (b) size distribution, (c) C1s high-resolution spectrum.” New Reference 32 will appear in the original publication as following: Siriwardena, A.; Khanal, M.; Barras, A.; Bande, O.; Mena-Barragan, T.; Ortiz Mellet, C.; Garcia Fernandez, J.M.; Boukherroub, R.; Szunerits, S. Unprecedented inhibition of glycosidase-catalyzed substrate hydrolysis by nanodiamond-grafted O-glycosides. *RSC Adv.* **2015**, *5*, 100568–100578. With this correction, the order of some references has been adjusted accordingly. 

**Figure 2:**
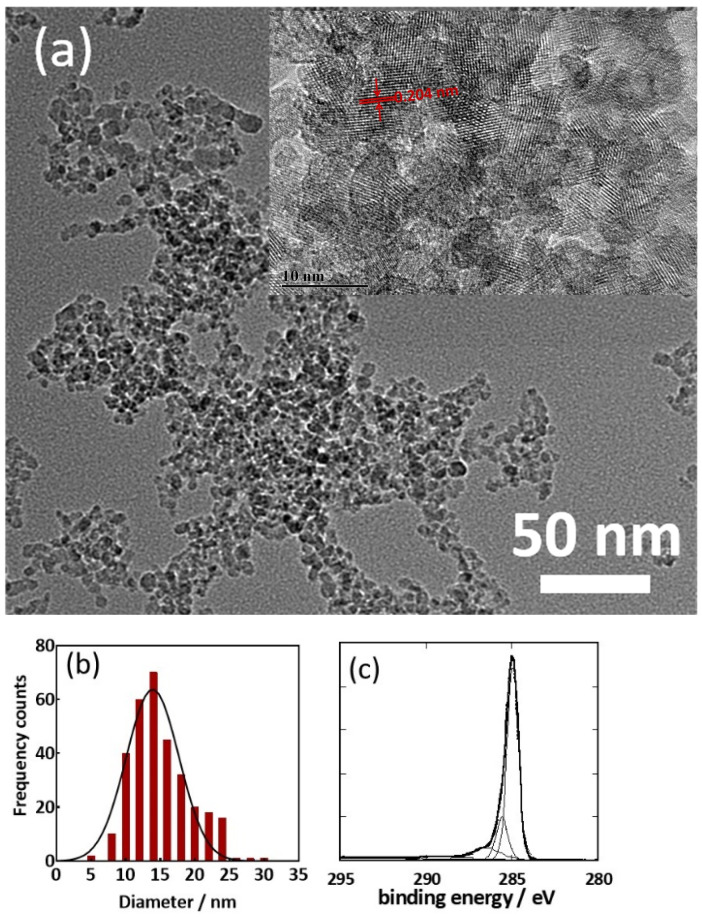
Characterization of nanodiamond particles (NDs) used in this work: (**a**) TEM (adapted with permission from ref. [32], 2015, Royal Society of Chemistry) and HR-TEM (inset), (**b**) size distribution, (**c**) C1s high-resolution spectrum.

The authors apologize for any inconvenience caused and state that the scientific conclusions are unaffected. The original publication has also been updated. This correction was approved by the Academic Editor.
